# Cueing-assisted gamified augmented-reality gait-and-balance rehabilitation at home for people with Parkinson’s disease: protocol of a pragmatic randomized controlled trial implemented in the clinical pathway

**DOI:** 10.3389/fneur.2025.1512409

**Published:** 2025-02-24

**Authors:** Daphne J. Geerse, Eva M. Hoogendoorn, Pieter F. van Doorn, Jara S. van Bergem, Annejet T. van Dam, Lotte E. S. Hardeman, Melvyn Roerdink

**Affiliations:** Department of Human Movement Sciences, Faculty of Behavioural and Movement Sciences, Vrije Universiteit Amsterdam, Amsterdam Movement Sciences, Amsterdam, Netherlands

**Keywords:** augmented reality, gamified exercise, cueing, Parkinson’s disease, clinical feasibility, effectiveness

## Abstract

**Background:**

Physiotherapy in the clinic is highly recommended for improving gait, balance, and fall risk in people with Parkinson’s disease. In addition, technology may help boost unsupervised exercise hours at home. Strolll is an augmented-reality (AR) neurorehabilitation platform for delivering gait-and-balance exercises onto AR glasses that can be performed under direct supervision of the therapist in the clinic, but also independently at home. Strolll AR also has the option to integrate AR cueing in gait-and-balance exercises to assist people with more severe mobility impairments in performing the exercises. The objective of this pragmatic randomized controlled trial (RCT) on Strolll AR is to examine its clinical feasibility and effectiveness for improving indicators of gait, balance, and falls risk. A secondary objective is to evaluate procedures for tailoring assistive AR cues.

**Methods:**

A total of 100 people with Parkinson’s disease (Hoehn and Yahr stages 1–3) with gait and/or balance impairments will participate in this study. This study is a pragmatic RCT in which all participants follow the same procedure. After a baseline assessment (T0), participants will start with a 6-week usual care control period, followed by a midterm assessment (T1). Subsequently, participants will undergo 2 weeks of in-clinic familiarization with Strolll AR. Then, participants will start with the 6-week Strolll AR intervention at home, followed by a final in-clinic assessment (T2). The primary study parameters are feasibility (i.e., safety, adherence, performance, and user experience) and effectiveness for improving indicators of gait, balance, and falls risk. For the statistical analyses on effectiveness, participants will be allocated to control (using T0-T1 change data) or intervention (using T1-T2 change data) groups using multiple (*n* = 20) randomizations. Recruitment started in May 2024 and the last T2 assessment is expected in February 2025.

**Discussion:**

The design of this particular pragmatic RCT will demonstrate feasibility and effectiveness in a real-world setting and in a representative population. Strolll AR may facilitate the transition from supervised care in the clinic to independent care at home, providing a platform for delivering individualized treatment, assisted with AR cues when deemed beneficial, for improving gait, balance, and falls risk in people with Parkinson’s disease.

**Clinical trial registration:**

https://clinicaltrials.gov/, identifier NCT06590987

## 1 Introduction

People with Parkinson’s disease face motor symptoms that limit activities of daily life (1–3), restrict participation (1, 2), and elevate falls risk (4). Physiotherapy for people with Parkinson’s disease aims to maximize quality of movement, functional independence, and general fitness, and to minimize secondary complications while supporting self-management and participation, and optimizing safety (5). It addresses core aspects like physical capacity, transfers, posture, upper-limb function, (in)activity utilizing cueing strategies, balance (and falls), and gait (2, 5), with balance and gait impairments often being indicators for an elevated falls risk (4). People with Parkinson’s disease often have a sedentary lifestyle (6), which may further worsen motor symptoms and associated falls risk. Physiotherapy, including prescribed unsupervised exercises at home, is highly recommended for improving gait, balance, and falls risk (1, 2, 7). That is, guidelines specific for people with Parkinson’s disease recommend to exercise for at least 150 minutes per week at a moderate to vigorous intensity, which can include (brisk) walking, balance training, and strength exercises (1, 7, 8), tailored to the specific needs of the person with Parkinson’s disease for the best outcome (9). However, this is often a difficult exercise target to reach and monitoring of the adherence often relies on self-reporting (10).

**Figure 1:**
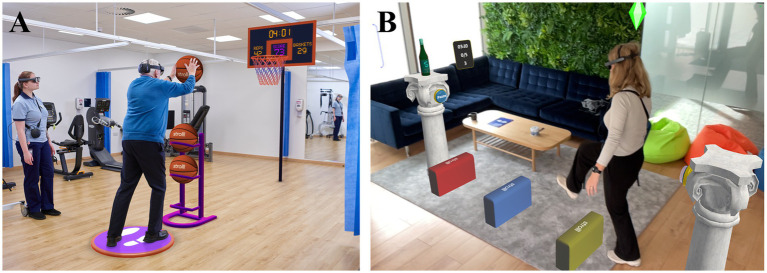
**(A)** Example of a Strolll AR gamified gait-and-balance exercise (i.e., Basketballl) performed in the clinic under the supervision of a therapist. **(B)** Example of a Strolll gamified gait-and-balance exercise performed independently at home (i.e., Smash!) complemented with assistive AR cues.

Emerging technology may help boost unsupervised exercise adherence at home (11). So far, most of these innovations, such as virtual-reality or screen-facing solutions have demonstrated promise in improving patient engagement and mobility outcomes (12–14). However, these technologies primarily focus on immersive or static experiences rather than facilitating interaction with a real-world environment. A promising technology in that regard is Strolll, an augmented-reality (AR) neurorehabilitation platform for delivering gait and balance exercises for Parkinson’s disease onto state-of-the-art AR glasses^1^ like Magic Leap 2 and Microsoft HoloLens 2. Various gamified and structured therapeutic exercises have been co-designed with people with Parkinson’s disease, therapists, and other stakeholders for gait and balance practice, aimed at obtaining engaging, effective therapy at high adherence (15). The Strolll AR platform comprises seven AR exercises that can be performed under direct supervision of the therapist in the clinic (Figure 1A), but also independently by people with Parkinson’s disease at home (Figure 1B), remotely prescribed and tailored by their therapists. Recently, Hardeman et al. (15) examined in a waitlist-controlled trial the feasibility and potential efficacy of Reality DTx^®^, the precursor of Strolll AR, for improving gait, balance, and falls risk in people with Parkinson’s disease in a research setting. This 6-week remotely prescribed home-based AR program was safe, adherable, usable, and well-accepted, with targeted intervention effects for improving indicators of gait, balance, and falls risk (15). Building on these promising results, obtained in a research setting, the current study aims to evaluate the feasibility and effectiveness of Strolll AR implemented in the clinical pathway, where therapists from more than 10 physical or exercise therapy clinics will integrate the Strolll AR platform with usual care, starting supervised in the clinic and then performed independently at home by people with Parkinson’s disease.

New to the Strolll AR platform is the option to integrate AR cueing in gait-and-balance exercises, which will assist people with more severe mobility impairments, like freezing of gait and festination, in performing the exercises. Cueing in the form of spatial (like visual lines or stepping targets) or temporal (like an acoustic rhythm) external stimuli aids the initiation, facilitation or modification of gait, generally with immediate effect (16–18). The effects of AR cueing delivered on state-of-the-art AR glasses with large vertical AR fields of views like Microsoft HoloLens 2 and Magic Leap 2 is not too dissimilar anymore from traditional cueing options. For example, our previous work showed that AR cueing alleviated freezing of gait in people with long and/or many freezing episodes (19) and that the gait-modifying effects of AR cueing were similar to those of real-world cueing [e.g., in terms of step-length and walking-speed modulation; (20)]. As cueing is not a one-size-fits-all solution (21, 22), the AR cues in the current study will be tailored to the individual’s preferences (e.g., some people benefit from 3D cues to step over, others from 2D cues to step onto (22, 23)) and gait characteristics (e.g., intercue distances fitting a person’s step length and width) in an attempt to yield optimal cueing effects during Strolll AR gait-and-balance exercises.

The objective of this pragmatic randomized controlled trial (RCT) on Strolll AR, an individualized AR gait-and-balance exercise platform complemented with tailored AR cues when deemed beneficial, is to examine its clinical feasibility and effectiveness. We expect that Strolll AR is (i) clinically feasible in terms of safety, adherence, performance, and user experience (i.e., for both people with Parkinson’s disease and their therapists) and (ii) effective for improving indicators of gait, balance, and falls risk in people with Parkinson’s disease. The secondary objective of this study is to evaluate procedures for tailoring assistive AR cues to individuals when deemed beneficial in performing the AR gait-and-balance exercises. We expect that many of the participants, particularly those with more severe mobility impairments like freezing of gait and festination, could benefit from assistive AR cues integrated in the AR exercises and that they use a variety of cue settings, thereby substantiating the necessity to personalize AR cues.

## 2 Methods and analysis

### 2.1 Study design

**Figure 2:**
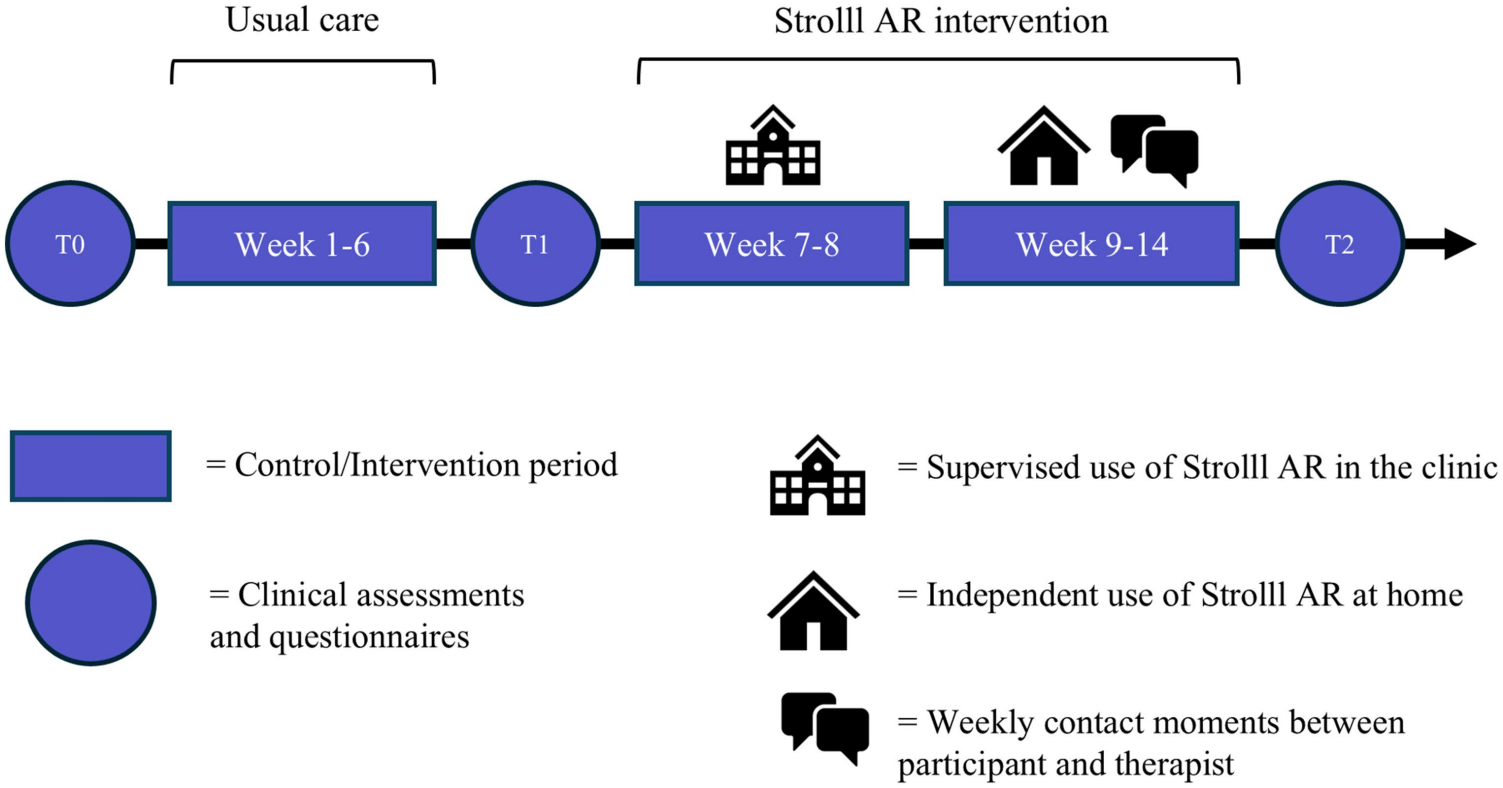
Schematic of the study design.

This study is a fully blinded two-armed pragmatic RCT, which will be executed in the Netherlands implemented at multiple physical or exercise therapy clinics. The study consists of three in-clinic assessments performed by participants’ own therapists (Figure 2). After a baseline assessment (T0), participants will start with a 6-week usual care control period, followed by a midterm assessment (T1). Subsequently, participants will undergo 2 weeks of in-clinic familiarization and training with Strolll AR supervised by their therapist. Then, participants will start with the 6-week Strolll AR intervention at home integrated with usual care, followed by the final in-clinic assessment (T2).

**Figure 3:**
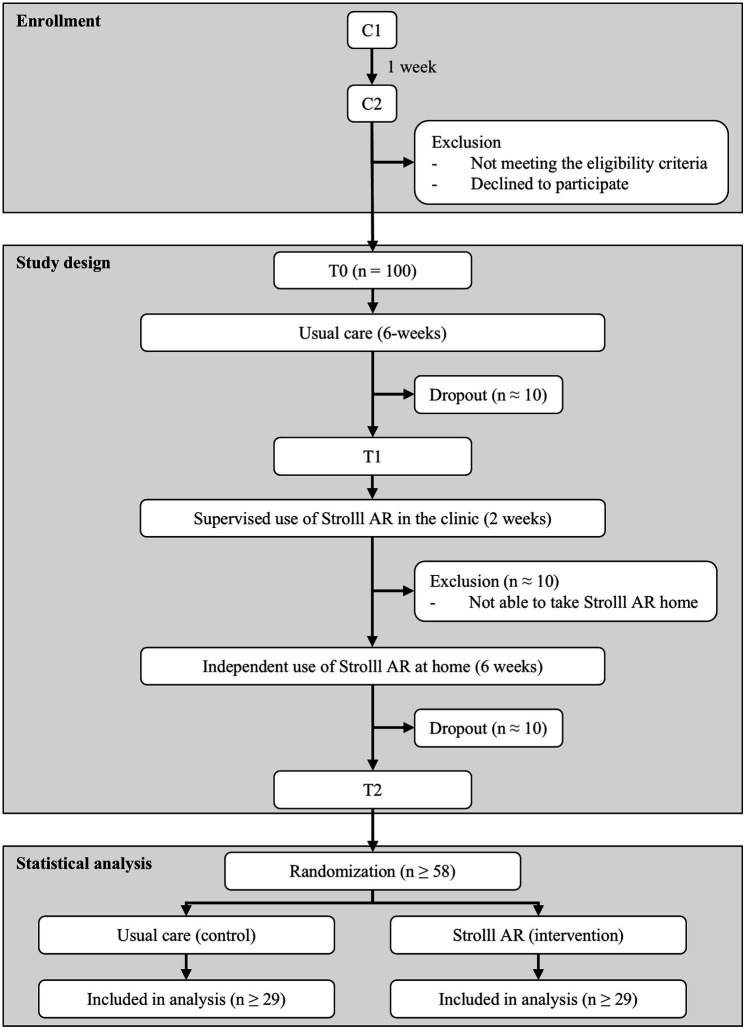
Flowchart from enrollment to statistical analysis.

The definition of a pragmatic RCT is that the intervention is tested in a real-world population in a real-world setting (i.e., the clinic), with an appropriate comparison arm (i.e., usual care alone in the current study) and relevant outcomes to determine its effectiveness (24, 25). Special trial designs can be considered. In this particular pragmatic RCT, all participants follow the same T0-control-T1-intervention-T2 procedure, suitable for both between-groups and within-subjects comparisons of the Strolll AR intervention effects as well as for evaluating its clinical feasibility. With regard to the between-groups comparison, participants will be allocated to control (using T0-T1 change data) or intervention (using T1-T2 change data) groups using multiple (*n* = 20) two-armed randomizations following a 1:1 ratio (Figure 3). The randomization will be predefined by an independent person using a Matlab (26) script (see Supplementary material S5), locked with a date and timestamp, and kept concealed from participants, therapists, and researchers. After the final T2 assessment of the last participant, the 20 randomizations of participants over control and intervention groups will be revealed to the researchers and applied in the data analysis (as detailed in section 2.7.3). Participants, therapists, and researchers will therefore all be blind to group allocation during the study, which is impossible for rehabilitation interventions with traditional RCT or cross-over designs. Note that a longitudinal design with a fixed order for all participants may be prone to time-dependent confounding factors, such as progressive worsening of Parkinson’s disease symptoms over time. We would like to emphasize that potential time effects associated with disease progression work conservatively against finding intervention effects. This particular pragmatic RCT design is not recommendable for situations where conditions improve over time (e.g., natural recovery after acute stroke), as this would promote arbitrary intervention effects associated with natural improvement in conditions over time. This design, in which all participants receive the Strolll AR intervention, further allows for a complementary within-subjects evaluation of the intervention effect (i.e., being less susceptible to between-subjects variation than aforementioned between-groups evaluation), while at the same time providing ample information on the clinical feasibility of the Strolll AR platform implemented in the clinical pathway in terms of its safety, adherence, performance, and user experience (i.e., from both participants’ and therapists’ perspectives), making efficient use of scarcely available resources (e.g., funding limiting scientific staff, time limiting availability of clinical staff, patients limiting the number of participants). Moreover, a design with the same procedure for all participants makes implementation of this study in the clinical pathway easier (hence the term pragmatic RCT) and potentially reduces the dropout rate because no participants are withheld from receiving the intervention due to the randomization.

### 2.2 Procedure and outcomes

After enrollment, the study consists of three in-clinic assessments spread out over 14 weeks, demarcating a usual-care control period and a Strolll AR intervention period (Figure 2). Below we describe the procedure for each of these parts of the design.

#### 2.2.1 Participant enrollment (C1, C2): enrollment and informed consent

The therapist will inform eligible participants about the study. If people are interested, they will receive a flyer with instructions on how to send their contact details to the researchers. Upon receival of contact details of a potential participant, the researcher will call this person to provide more information about the study (C1, Figure 3). Hereafter, potential participants will receive the participant information letter by mail or email. A week after receiving the participant information letter, the researcher will again call the potential participant (C2, Figure 3) to answer any questions and to ask if he or she wants to participate. If so, in- and exclusion criteria will be checked by the researcher (to the extent possible, otherwise criteria will be evaluated during the baseline assessment session T0). Furthermore, the potential participant will be informed that the medication should remain stable during the study. If the potential participant is eligible, he or she will be asked to sign the informed consent form (see Supplementary material S1), after which the researcher also signs the informed consent form and notifies the therapist that the person can start with the pragmatic RCT. Written informed consent must be given before commencing with the baseline assessment (T0).

#### 2.2.2 Baseline assessment (T0): characterization and effectiveness

The baseline assessment serves to characterize the study sample and to provide baseline values for evaluating the effectiveness of Strolll AR (see also Table 1). Study parameters used to characterize the participants are demographics (i.e., age, gender, disease duration) and current medication use [i.e., the Levodopa Equivalent Daily Dose (LEDD)]. Questionnaires to describe the participant population are the Montreal Cognitive Assessment [MoCA; (27), lower scores reflect cognitive decline], Movement Disorders Society Unified Parkinson Disease Rating Scale [MDS-UPDRS; (28), higher scores reflect more severe impairment], New Freezing of Gait Questionnaire [NFOGQ; (29), non-zero scores define freezers], Physical Activity Scale for the Elderly [PASE; (30), higher scores reflect greater self-reported physical activity], and 12-month falls history. All these outcomes will be collected using online questionnaires, except for the MoCA and MDS-UPDRS part Ia, III, and IV, which will be administered in the clinic by the therapist. To be able to evaluate the potential effects of Strolll AR for improving indicators of gait, balance, and falls risk, the following standard clinical tests will be administered by the therapists: Timed Up-and-Go test [TUG; (31), in seconds; a faster completion time reflects better performance], Five Times Sit-to-Stand test [FTSTS; (32), in seconds; a faster completion time reflects better performance], 10-meter walk test [10MWT; (33), in seconds; a faster completion time reflects better performance] and Mini Balance Evaluation Scale Test [(Mini-BESTest; (34), higher scores reflect better performance]. In addition, participants will fill out the Falls Efficacy Scale International [FES-I; (35), higher scores reflect higher concern of falling].

**Table 1:** Overview of T0, T1, and T2 tests and questionnaires to characterize the study sample and to evaluate the effectiveness and clinical feasibility of the Strolll AR intervention.

	T0	T1	T2
Study sample characterization			
Demographics (age, gender, disease duration)	X		
LEDD	X		
MoCA	X		
MDS-UPDRS	X		
NFOGQ	X		
PASE	X		
12-month falls history	X		
Effectiveness			
TUG*	X	X	X
FTSTS	X	X	X
10MWT	X	X	X
Mini-BESTest	X	X	X
FES-I	X	X	X
Clinical feasibility			
Falls history during usual-care and intervention periods		X	X
Strolll AR evaluation questionnaire			X
UEQ			X
Technology acceptance and use questionnaire			X
Top-3 barriers and facilitators of Strolll AR			X

LEDD, Levodopa Equivalent Daily Dose; MoCA, Montreal Cognitive Assessment; MDS-UPDRS, Movement Disorders Society Unified Parkinson Disease Rating; NFOGQ, New Freezing of Gait Questionnaire; PASE, Physical Activity Scale for the Elderly; TUG, Timed Up-and-Go test; FTSTS, Five Times Sit-to-Stand test; 10MWT, 10-meter walk test; Mini-BESTest, Mini Balance Evaluation Scale Test; FES-I, Falls Efficacy Scale International; UEQ, User Experience Questionnaire. *Primary outcome measure.

#### 2.2.3 Usual care (6-weeks)

During the usual-care control period, participants will not receive any additional training or instructions related to the Strolll AR intervention. In line with the pragmatic nature of the trial, participants will, however, continue with their weekly therapy schedule with their therapist, as provided prior to the study. The therapists involved are affiliated with the ParkinsonNet network (Dutch society for Parkinson’s therapists (36) and deliver therapy following the Dutch physiotherapy guidelines for Parkinson’s disease (1).

#### 2.2.4 Midterm assessment (T1): effectiveness

For the midterm assessment, the standard clinical tests of gait, balance, and falls-risk indicators (TUG, FTSTS, 10MWT, Mini-BESTest) (Table 1) will again be administered by the therapist. In addition, the participants are asked to fill out the FES-I and 6-week falls history (to assess falls during the usual-care period) questionnaires at home (Table 1).

#### 2.2.5 Practice with Strolll AR intervention supervised in clinic (2 weeks)

To make the participant familiar with the Strolll AR platform technology and procedures and to allow the therapist to evaluate safety aspects, the Strolll AR intervention starts with 1 to 3 supervised in-clinic sessions of (cueing-assisted) personalized AR exercises.

In these supervised sessions in the clinic, the therapist will also test and validate procedures to select and tailor effective assistive AR cues from a library of different AR cues (see Supplementary material S2 for the types of cues used in this study). This is needed for two reasons: (1) to test the premise that a one-size-fits-all cue does not exist as there is heterogeneity in the effect of cues among participants, (2) to ensure that the Strolll AR gait-and-balance exercises can be effectively assisted with personalized cues for those who need it (e.g., people experiencing freezing of gait or people with small steps and shuffling gait (16–18)). The so-obtained best cues for supporting gait, as evaluated subjectively using both the participant’s experience and the therapist’s impression, will be used during the remainder of the Strolll AR intervention for those who are deemed to benefit from it. We will document if AR cues are added to Strolll AR gait-and-balance exercises, and when applicable, specify the type of AR cues as well as their personalized parameters (intercue distance, height, width, audiofeedback, etc.).

To deliver the Strolll AR intervention, participants in this study will by default be equipped with Magic Leap 2 AR glasses for its unmatched vertical AR field of view. Special insert lenses from a complementary lens kit (supports 8 prescriptions from −2.0 to +3.0) made available at each clinical site will be used to correct vision for people wearing spectacles. In case vision cannot be corrected with these insert lenses (e.g., due to a special prescription not part of the lens kit), either tailored insert lenses will be ordered for Magic Leap 2 or Microsoft HoloLens 2 with a slightly smaller vertical AR field of view will be used which can be worn over spectacles to deliver the intervention.

Once the participant is deemed sufficiently capable of using the Strolll AR platform technology independently at home in a safe manner (after training in the clinic with the therapist for one to maximally three sessions of 1 hour in 2 weeks), the participant will take the AR glasses home together with a complementary SIM-card equipped WiFi router to continue the Strolll AR intervention independently at home. If the Strolll AR intervention at home is not deemed safe or suitable by the therapist, the intervention will stop and the reason for this will be documented by the therapist as part of the clinical feasibility evaluation.

#### 2.2.6 Training with Strolll AR intervention independently at home (6 weeks)

**Figure 4:**
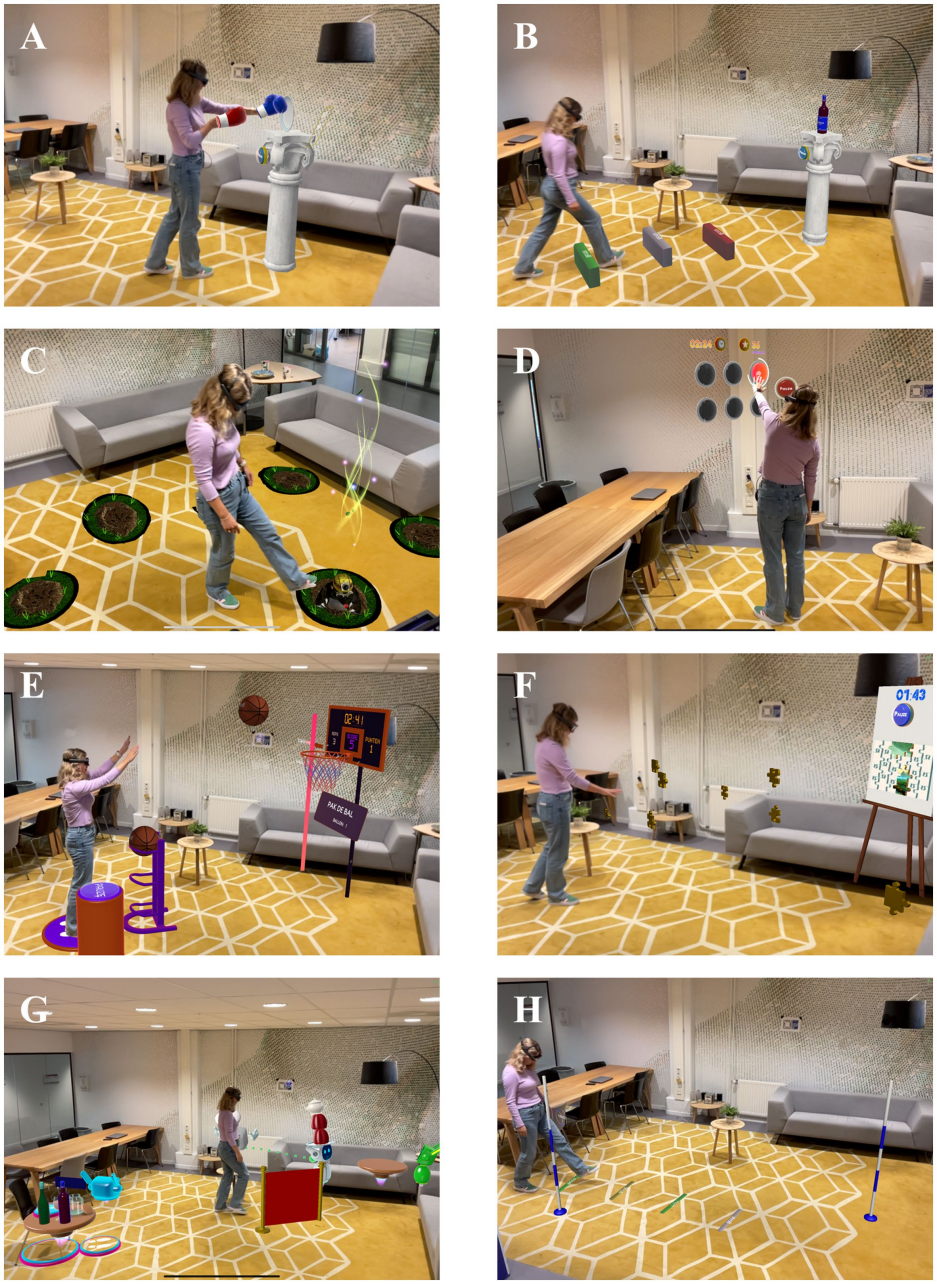
Strolll AR gait-and-balance exercises: Smash! [without **(A)** and with **(B)** assistive AR cues], Mole Patrolll **(C)**, Hot Buttons **(D)**, Basketballl **(E)**, Puzzle Walk **(F)**, Wobbly Waiter **(G)**, and Cue Challenge **(H)**.

The Strolll AR intervention, integrated with usual care, comprises of a 6-week home-based individualized AR gait-and-balance exercise program assisted with tailored AR cues when deemed beneficial. The AR exercises are designed to train aspects associated with gait, balance, and falls risk in a gamified manner to maximize training compliance. Strolll AR comprises seven AR exercises, namely Basketballl, Smash!, Mole Patrolll, Hot Buttons, Puzzle Walk, Wobbly Waiter, and Cue Challenge (Figure 4; see Supplementary material S2 for a detailed description of all AR exercises), intended to improve gait, balance, and falls risk in people with Parkinson’s disease. Exercises that require walking have optional complementary AR cueing (Smash!, Cue Challenge), integrated cueing (speed cue in Wobbly Waiter) or AR-mediated goal-directedness (the moles in Mole Patrolll, the puzzle pieces in Puzzle Walk), to various degrees enabling and assisting people with more severe mobility impairments to participate. For each AR exercise, feedback of performance in terms of game-play performance metrics and functional performance metrics are given during the exercises. For Basketballl, for example, metrics associated with the number of balls thrown in the basket (game-play performance) and the number of sit-to-stands or squats made (functional performance) are given, derived from AR glasses data. See Supplementary material S2 for more details on the AR exercises, metrics, and types of cues.

**Figure 5:**
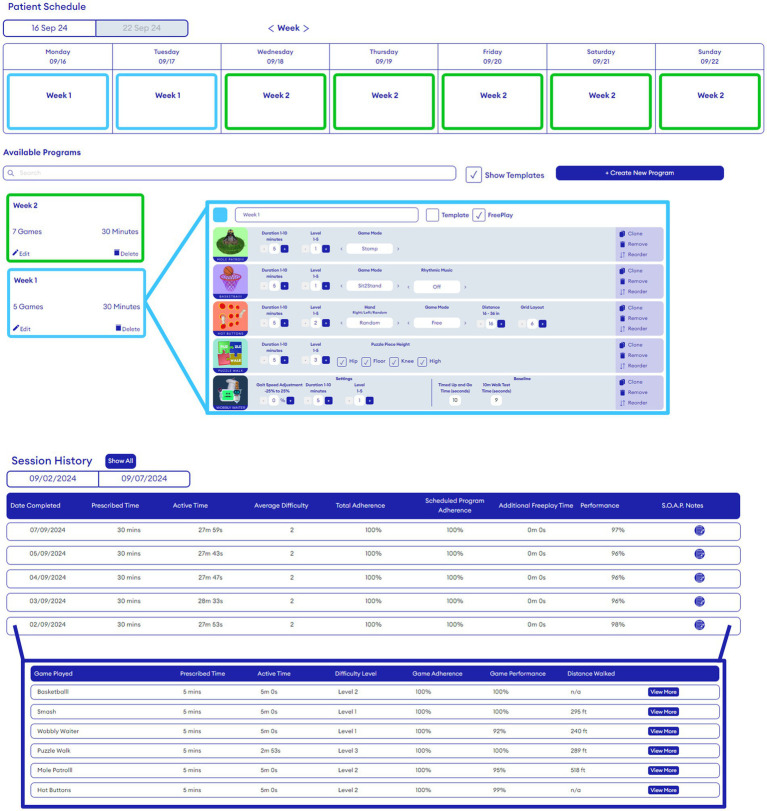
Strolll AR web portal for therapists to (remotely) prescribe individualized treatment programs (type and settings of AR exercises, top part) and to see feedback on adherence and game-play performance (session history, lower part).

Participants will be invited by their therapist to use Strolll AR minimally 5 times a week for 30 active minutes per day, resulting in the recommended 150 minutes per week (1, 7, 8). They can perform the exercises anytime during the day, in one bout or in multiple chunks during the day, with complementary AR cueing when deemed beneficial. The precise exercises, exercise duration, difficulty level, and order will be prescribed by the therapist via the Strolll web portal (Figure 5). Therapists were by default recommended to prescribe all AR exercises, as each AR exercise targets different aspects (see Supplementary material S2). However, they were allowed to make adjustments, if necessary, to align with the participant’s abilities and needs, as well as for practical considerations. The prescribed exercises will be evaluated and adjusted on a weekly basis in telephone calls, e-consults or during an onsite therapy session using information of the participant in combination with the feedback from exercises that are reported in the Strolll web portal, such as adherence and game-play and functional performance metrics (cf. Supplementary material S2). Adherence will be defined as the percentage of performed over prescribed gait-and-balance exercises [i.e., both frequency and session-duration adherence; (15)]. The data on game-play level and performance will be used to evaluate whether the exercise program was both progressive (increase in level) and achievable (consistently high game-play performance scores) as intended. The weekly contact moments with the therapist will also be used to ask if participants experienced any adverse events (safety flags). Specifically, safety will be evaluated as the number of adverse events due to the Strolll AR intervention, such as falls, dizziness, eye strain, and headache. Potential technical issues will be documented by the researcher, who will be available to provide technical support during the study based on email or telephone contact with the participants.

#### 2.2.7 Final assessment (T2): effectiveness and clinical feasibility

To be able to evaluate the effectiveness of the Strolll AR intervention, the therapist will again administer standard clinical tests of gait, balance, and falls risk (TUG, FTSTS, 10MWT, Mini-BESTest). Participants will fill out the FES-I questionnaire together with the researcher during a telephone call (Table 1).

Clinical feasibility of the Strolll AR platform according to the participants will be assessed in terms of user experience [i.e., the User Experience Questionnaire (UEQ); (37)], technology acceptance and use [i.e., questionnaire based on the Unified Theory of Acceptance and Use of Technology model (UTAUT; (38))], top-3 barriers and facilitators of Strolll AR, and intervention-specific questions regarding Strolll AR, including cueing if used during the intervention (see Supplementary material S3). Participants will also report their falls history during the intervention period. The questionnaires of the final assessment are completed through a telephone call with the participant to ensure comprehensibility, which was first assessed in think-aloud sessions with a number of participants (39). In light of the potential future implementation in the clinical pathway, therapists will also be asked about their experience with Strolll AR through a purpose-made questionnaire (see Supplementary material S4), including the technology acceptance and use questionnaire, the top-3 barriers and facilitators (assessed before training their first participants and after training their last participant), and the UEQ (assessed after their last participant completed the study). Furthermore, a focus group with the therapist will be organized after the study for an in-depth evaluation of the top-3 barriers and facilitators.

### 2.3 Participants

We aim to include 100 people with Parkinson’s disease in this pragmatic RCT. To be eligible to participate, a person must meet the following criteria:

- 21 years or older
- have command of the Dutch language
- diagnosed with Parkinson’s disease according to the UK Parkinson’s Disease Brain Bank criteria (stages 1–3 on the Hoehn and Yahr scale)
- bothersome gait or balance impairments (i.e., negatively affecting their ability to perform their usual daily activities as indicated by the person with Parkinson’s disease and/or their therapist).

A potential participant who meets any of the following criteria will be excluded from participation:

- inability to comply with the protocol, i.e., additional neurological diseases and/or orthopedic problems seriously interfering with gait function
- insufficient physical capacity to safely perform the intervention (e.g., frequent faller) or severe cognitive impairments (as observed by the therapist)
- visual or hearing impairments (after corrective aids)
- inability to walk independently for 30 min (in bouts of 5–10 min)
- severe visual hallucinations or illusions
- no stable dosage of medication.

Finally, in case potential participants are susceptible to hallucinations, have a history of seizures, have deep brain stimulation, or a pacemaker, it should be discussed with their therapist if they can participate, as per Strolll’s Instructions For Use (DOC-IFU-00021 (NL), Revision 01, Date of Issue 062024). Participants are not allowed to participate in other intervention studies during the study.

Participants will be recruited by their own therapist (see the flowchart in Figure 3 for the procedure). Approximately 10 physical or exercise therapy clinics will participate, recruited via regional ParkinsonNet meetings and using a recruitment text. Clinics will be primary care practices that have therapists affiliated with ParkinsonNet [Dutch society for Parkinson’s therapists (36)]. Only clinics that indicate that they have the capacity to include minimally 5–10 participants within 6–9 months will be included. Therapists will be trained on how to administer the clinical tests (they are already familiar with most if not all tests as these are typically used tests in clinical practice) to ensure a high interrater reliability, and in applying the in- and exclusion criteria for the selection of eligible participants. In addition, therapists will be trained by the researchers on how to use the AR glasses, the Strolll AR application, and the Strolll web portal for remote prescription of and feedback about the Strolll AR intervention. After this initial training, therapists will take the AR glasses home to practice and become familiar with both the Strolll AR application and the Strolll web portal themselves. After practicing for 1–2 weeks, the researchers will contact the therapist again to discuss the use of the Strolll AR platform and to offer support during the first in-clinic session with Strolll AR with a participant to ensure therapists are well-versed with the study protocol and the Strolll AR platform. Recruitment started in May 2024 and the last T2 assessment is expected in February 2025.

### 2.4 Sample size calculation

A convenience sample of 100 participants will be included and measured from approximately 10 clinics in a time frame of 9 months. This sample size will give the therapists of the clinics enough experience with the Strolll AR platform and intervention to evaluate feasibility from a therapists’ perspective (e.g., usability, acceptability, safety). For our effectiveness outcomes, an a-priori required sample-size calculation in G*Power 3.1.9.7 showed that 58 participants (i.e., 29 per group) is sufficient for identifying statistically significant between-group differences (usual care vs. Strolll AR intervention). This is based on 80% power and a two-tailed alpha error of 5% and a minimal improvement of 2.57 s in TUG [i.e., the average of the smallest detectable TUG difference of Lim et al. (40) and Huang et al. (41)] and an effect size of 0.755 [Cohen’s *d* statistic; SD of 3.4 s; (41)]. Accounting for approximately 10 dropouts during the usual-care period, approximately 10 exclusions during the pre-intervention in-clinic phase (i.e., participants deemed unable to take the glasses home after one-to-three in-clinic training sessions) and approximately 10 dropouts during the intervention period at home [estimates based on Hardeman et al. (15)], recruiting a total of 100 participants should be sufficient for obtaining the minimal groups sizes required for identifying between-group differences. As dropout rates are taken into account, participants who are not completing the study will not be replaced. Plans to reduce dropouts are a voucher as a token of appreciation for their participation, updates about study progression via a newsletter send to participants and therapists every 2 months to keep them involved in the study and having weekly online or onsite check-ins with the participating therapists.

### 2.5 Discontinuation or modification of allocated intervention

Participants can leave the study at any time for any reason if they wish to do so without any consequences. The researcher can decide to withdraw a participant from the study for urgent medical reasons or when medication dosage is changed during the study. Participants that are withdrawn will not be replaced, because the sample-size calculation takes such dropouts into account. Reasons for drop out or withdrawal will be collected. Dropouts during the intervention period will be asked to complete the UEQ and Strolll AR evaluation questionnaire to reduce potential bias in the study’s participant experience results which could become manifest if only data from participants completing the intervention would be taken into account. For the other clinical tests and questionnaires, dropouts will lead to missing data.

### 2.6 Premature termination of the study

The study will be terminated prematurely if serious events, like falls that lead to hospitalization, related to the Strolll AR intervention at home are reported for more than two participants. A liability insurance is in place in accordance with the legal requirements in the Netherlands, specifically article 7 of the Medical Research Involving Human Subjects Act (in Dutch: Wet Medisch-wetenschappelijk Onderzoek met Mensen, WMO). This insurance provides cover for damage to research participants through injury or death caused by the study. The insurance applies to the damage that becomes apparent during the study or within 4 years after the end of the study.

### 2.7 Statistical analysis

Data analysis will be performed in JASP (42). Missing data will be excluded analysis-by-analysis.

#### 2.7.1 Group characterization

Descriptive statistics of demographics (i.e., age, gender, disease duration, and LEDD) and questionnaires and clinical tests (i.e., MoCA, MDS-UPDRS, NFOGQ, PASE, and falls history) will be used to characterize the study sample (*n* = 100). To identify potential differences between control and intervention groups after the randomizations, independent-samples *t*-tests will be used after confirming normality with Shapiro–Wilk tests (otherwise Mann–Whitney *U*-tests will be used) for all parameters except gender, for which a Chi-square test will be used to identify between-groups differences.

#### 2.7.2 Feasibility (safety, adherence, performance, and user experience)

Clinical feasibility will be evaluated on several aspects. First, to determine if the Strolll AR intervention can be safely performed at home, descriptive outcomes will be reported for safety (i.e., number of dropouts during training at home including the reasons for withdrawal, adverse events including a paired-samples *t*-test comparison of the average weekly number of falls experienced during usual-care and intervention periods), providing insight into the potential risks of the Strolll AR intervention.

Second, to determine if the Strolll AR intervention was adherable over the course of the intervention, we will conduct a repeated-measures one-way ANOVA with the within-subject factor Time (three levels) on adherence scores derived from the first, second, and third part of the intervention at home (i.e., default intervention period is 6 weeks, so adherence is derived over roughly two-weeks intervals). The assumption of sphericity will be checked according to Girden (43). If Greenhouse–Geisser’s epsilon exceeds 0.75, the Huynh-Feldt correction will be applied; otherwise, the Greenhouse–Geisser correction will be used. Effect sizes will be quantified with *η*_p_^2^. Paired-samples *t*-tests will be used for post-hoc comparisons of a significant main effect of Time. In addition, one sample *t*-tests against 100% will be performed for adherence scores at each Time point to determine whether or not there were systematic deviations from the prescribed treatment.

Third, the pre-cursor of Strolll AR, Reality DTx^®^, was found to be a progressive-but-achievable intervention in a research setting (15), where researchers balanced and tailored task demands and capacity (i.e., not too easy to prevent boredom and not too difficult to prevent demotivation) when remotely prescribing the exercises based on weekly telephone calls with the participants and their performance data from the Strolll web portal. To determine if Strolll AR, delivered in the clinical pathway, also results in a progressive-but-achievable intervention (i.e., increasing exercise levels over weeks while maintaining high-performance scores), levels and performance scores for each game were subjected to repeated-measures ANOVAs (or their non-parametric equivalent) with the factor Time (3 levels, first, middle, last part), using paired-samples *t*-tests (or their non-parametric equivalent) post-hoc analyses for significant effects of Time. We expect to find strong main effects of Time for exercise levels (with post-hoc effects showing an increase in exercise levels), but not or less so for performance scores.

Fourth, user experience will inform on acceptability among people with Parkinson’s disease and among therapists (i.e., top-3 barriers and facilitators questionnaire and an in-depth focus group) and usability (UEQ from both participants and therapists) of the Strolll AR intervention. To evaluate participant experience, all participants who started with the Strolll AR intervention in the clinic will be analyzed collectively (i.e., intention-to-treat protocol) to avoid a selection bias of the people who completed the intervention. The UEQ will not be analyzed statistically but will be reported as descriptive outcomes and interpreted against known benchmark scores (37). Also, other participants’ or therapists’ reported outcome measures from the questionnaires will be reported as descriptive outcomes. Finally, the fraction of participants deemed eligible for performing the intervention at home after having had the in-clinic Strolll AR sessions will inform about the usability of the intervention.

#### 2.7.3 Effectiveness

The effectiveness of Strolll AR for improving TUG, FTSTS, 10MWT, Mini-BESTest, and FES-I will be analyzed according to the per-protocol principle for participants who have completed the final assessment, randomized into control and intervention groups using the 20 concealed randomizations. For each outcome parameter, independent-samples *t*-tests (or their non-parametric equivalents) will be used to compare Control (i.e., using the change scores during the control period; T0-T1) and Intervention groups (i.e., using the change scores during the intervention period; T1-T2). We expect significantly higher change scores for the Intervention group than for the Control group. The 20 predefined concealed randomizations will enable us to perform multiple between-group comparisons, yielding a sensitivity analysis to test the robustness of the effectiveness findings in this pragmatic RCT. That is, this innovative analysis approach will not only provide us with a one-shot indication of the statistical effect, but also with a confidence interval of this effect. Furthermore, multiple randomized comparisons will reduce the likelihood of a systematic confounding effect biasing this pragmatic RCT results, as is often the case when a single one-shot randomization would result in Control and Intervention groups differing significantly at baseline in certain demographic or clinical characteristics. With a multiple-shots randomization, potential effects associated with significant between-group differences in demographics or clinical characteristics of a single randomization will average out over the multiple shots.

As a secondary analysis, which is possible given the design in which all per-protocol participants performed the same T0-control-T1-intervention-T2 procedure, a complementary within-subjects comparison will be performed using repeated-measures ANOVAs with the within-subject factor Time (three levels: T0, T1, T2) for the TUG, FTSTST, 10MWT, Mini-BESTest and FES-I, comparable to the analysis performed in Hardeman et al. (15). For significant main effects of Time, the first and second reverse Helmert contrasts will be used to evaluate Control and Intervention effects, respectively. This will help us interpret (a potential null-finding of) the pragmatic RCT as such a within-subjects comparison is less susceptible to between-subjects variation than a between-groups comparison.

## 3 Discussion

The objective of this pragmatic RCT on Strolll AR, an individualized AR gait-and-balance exercise platform complemented with tailored AR cues when deemed beneficial, is to examine its clinical feasibility and effectiveness for improving indicators of gait, balance, and falls risk in people with Parkinson’s disease. This study design will demonstrate feasibility and effectiveness in a real-world setting and for a population representative of people with Parkinson’s disease seen in the clinical pathway. The secondary objective of this study is to evaluate procedures for tailoring assistive AR cues to individual people with Parkinson’s disease when deemed beneficial for them in performing the AR gait-and-balance exercises.

The number of people with chronic neurological disorders like Parkinson’s disease waiting for physiotherapy treatment is increasing (44). Due to the growing number of people with a chronic neurological disorder and the limited number of staff available, digital therapeutics solutions emerge such as the Strolll AR platform. Such innovative technologies are well aligned with the mission statement of care in the Netherlands (45, 46), emphasizing the importance of (i) care in the home environment, if possible, (ii) independent care, if possible (self-management), and (iii) digitally supported care, if possible (supported by technology). The Strolll AR platform aligns well with this mission. It is a home-based intervention, which can be executed by the person with Parkinson’s disease themselves on AR glasses, remotely prescribed by a therapist. The gamified nature of Strolll AR, combined with the remote prescription and reporting options and data dashboards, also have the potential to increase the adherence to prescribed therapy at home, thereby likely improving treatment effectiveness. Finally, Strolll AR provides people with Parkinson’s disease the flexibility, independence, and ownership of their rehabilitation to be performed when they feel up for it (e.g., depending on fatigue and medication levels, which fluctuate during the day) in the convenience of their own home and work/life schedule, allowing for more professionally prescribed treatment sessions and/or treatment hours without the burden and costs associated with commuting to the healthcare professional for a session fitting their time schedule. Hardeman et al. (15) already showed that the precursor of the Strolll AR platform showed promising results in improving indicators of gait, balance, and falls risk. Moreover, this then-called Reality DTx® AR intervention was safe and deemed acceptable and usable by the participants. The current study builds on these findings, that were obtained in a research setting, by conducting a pragmatic RCT implemented in a representative clinical pathway.

For future implementation of Strolll AR, it is important to know, besides its effectiveness in a real-world clinical setting, what the stakeholders think of the intervention. Determining information about the clinical feasibility of Strolll AR, both from therapists’ and participants’ perspectives, is therefore a key focus of this study. Since the context wherein Strolll AR is evaluated in this study is similar to the clinical pathway for which Strolll AR is intended, the feedback from the questionnaires is representative and relevant for its further development and implementation after study completion. That is, safety, adherence, usability, and acceptability feedback might help to optimize the fit between state-of-the-art physiotherapy practice and the Strolll AR platform. Moreover, insights into top barriers and facilitators experienced by therapists will help in choosing the most pertinent implementation strategies (47).

Considering the pressing needs for transforming the future of healthcare, AR technology integrated in the clinical pathway of people with Parkinson’s disease seems one of the ways to go. It may help in combatting waitlists, in improving accessibility of care, in increasing the number of treatments as well as their adherence, and perhaps even in pivoting from healthcare to care for health, thereby capitalizing on the importance of high-quality exercise in maintaining health and preventing decline. Moreover, Strolll AR may be a platform that facilitates the transition from supervised care in the clinic to independent care at home. That is, if proven effective, Strolll AR could provide a platform for delivering individualized treatment at a high dose and adherence, improving gait, balance, and falls risk in people with Parkinson’s disease. As such it has the potential for bringing evidence-based rehabilitation into the homes of people with Parkinson’s disease (11), thereby fostering longer-term self-management of health and disease. Nevertheless, while technology like AR evolves at a rapid and accelerated pace, changes in clinical practice are typically slow and dependent on a solid evidence base. The innovative design of this pragmatic RCT, allowing for studying both the clinical feasibility and effectiveness in a single study design in representative real-world settings, could potentially help speed up the availability of the evidence required for the uptake of technology-driven interventions like Strolll AR in the clinical pathway.

### Data management and monitoring

Questionnaires and clinical tests collected in the clinic will be collected on paper and will be entered into a digital database (i.e., Castor EDC). Digital copies and an export of the digital database will be stored on a secured drive (i.e., Research Drive). Home questionnaires can also directly be entered into the digital database by the participants using an online questionnaire. Personal data will be handled according to the EU General Data Protection Regulation and the Dutch Act on Implementation of the General Data Protection Regulation. Personal data to contact participants will be stored separately from other data and provided with an ID number. An identification file with codes linking ID numbers to participant numbers (four-digit codes) will be stored in a folder separate from the contact details file with ID numbers. Files with personal data to contact participants and the identification file can only be accessed with a password. Data collected on paper will be stored in a secured cabinet. Administrative data (logbooks for data collection and analysis, manuals, protocols) will be stored on OneDrive to improve interpretation and re-use of the data. All digital drives and databases require multi-factor authentication. Monitoring will be executed by (internal) monitors according to the Netherlands Federation of University Medical Centres (NFU) guidelines as approved by the accredited Medical Ethics Committee United.

## Footnotes

^1^ www.strolll.co
