# Supplementary material for: Cueing-assisted gamified augmented-reality gait-and-balance rehabilitation at home for people with Parkinson’s disease: protocol of a pragmatic randomized controlled trial implemented in the clinical pathway
Source: Front Neurol. 2025 Feb 24;16:1512409. doi: 10.3389/fneur.2025.1512409 (PMC11891067; doi:10.3389/fneur.2025.1512409)
Supplement: Supplementary file 2 [file Table_1.docx]

Supplementary Material

# Supplementary Data Sheet 1. Informed consent form

**Informed consent form**

Related to

*Strolll AR: Cueing-assisted gamified augmented-reality gait-and-balance rehabilitation at home for people with Parkinson’s disease*

Team Holocue(X), Vrije Universiteit Amsterdam

- I have read the information letter. I was also able to ask questions. My questions have been adequately answered. I had enough time to decide whether I wanted to participate.
- I understand that participation is voluntary. I also know that I can withdraw or stop my participation at any time. I do not have to say why I want to stop.
- I know that I can withdraw my consent for the use of my data. This has no adverse consequences for me as a patient.
- I give the researcher permission to inform my GP that I am participating in this study.
- I give the researchers permission to collect and use my data, such as medication use, disease severity and results of standardized clinical gait and balance tests. The researchers do this only to answer the research question of this study (i.e. determining the clinical feasibility and effectiveness of Strolll AR).
- I know that, for the purpose of checking the quality of the research, some people can view all my data. These people are listed in the information letter. I give these people permission to view my data for this purpose.
- I will return the glasses with the Strolll AR exercise program to the researchers after the training.

- Please indicate ‘yes’ or ‘no’ in the table below.

| I give permission to store my data for use in other research, as described in the information letter. | Yes ☐ | No ☐ |
| --- | --- | --- |
| I give permission to ask me after this study if I want to participate in a follow-up study. | Yes ☐ | No ☐ |

- By signing I indicate that I want to participate in this study.

My name is (participant): ………………………………..

Signature: ……………………… Date : __ / __ / __

-----------------------------------------------------------------------------------------------------------------

I confirm that I have fully informed this participant about the study described above. If information emerges during the research that could influence the participant’s willingness to participate, I will inform this participant in a timely manner.

Researcher’s name (or representative):……………………………….

Signature:……………………… Date: __ / __ / __

# Supplementary Data Sheet 2. Strolll AR exercise and cues

| **Strolll AR exercise** | | | |
| --- | --- | --- | --- |
|  | **Description of the exercise** | **Exercise settings** | **Feedback** |
| **Smash!**    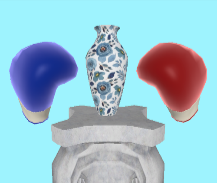 | A boxing rehabilitation exercise to train gait, dynamic balance, weight shifting and turning.    The goal is to smash as many items as possible from two plinths as they appear, demanding alternate left and right punches to promote weight shifting, with available items alternating between the plinths to promote walking and turning. | • Duration of the exercise (1 - 10 minutes)  • Difficulty level (number of required punches before the items drop from the plinth, 2 – 20 punches)  • Distance between the plinths (2 - 10 meters)  • Optional addition of cues between plinths (see Table S2 for more details) | *In-game feedback*  • Number of prescribed and performed punches  • Score (number of items smashed)    *Post-game feedback*  • Score    *Web portal feedback*  • Prescribed and active minutes  • Meters walked  • Number of punches  • Score |
| **Mole Patrolll**    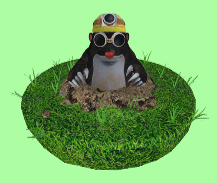 | A goal-directed walking rehabilitation exercise to train gait initiation, walking adaptability, dynamic balance, turning, stopping and strength (when performed in squat mode).    The goal is to stomp as many moles as possible by scanning the room, spotting where they appear, and stomping on them either with both feet or squatting on them (a game-mode setting) before they disappear. | • Duration of the exercise (1 - 10 minutes)  • Difficulty level (time before mole disappears, 1 – 60 seconds per mole)  • Game mode (stomp or squat mode) | *In-game feedback*  • Score (number of moles stomped)  • Distance walked    *Post-game feedback*  • Score    *Web portal feedback*  • Prescribed and active minutes  • Meters walked  • Score |
| **Hot Buttons**    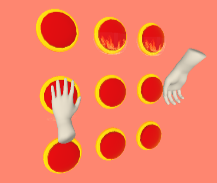 | A dynamic reaching exercise to train functional reaching, reaction time and dynamic balance.    The goal is to press the button that lights up as quickly as possible before it disappears. Feet positioning is controlled to ensure sufficient reach amplitudes. | • Duration of the exercise: (1 - 10 minutes)  • Difficulty level (time before button disappears, 1.5 – 60 seconds time out)  • Game mode (free standing, wall, table)  • Game mode (3, 6 or 9 buttons)  • Game mode (random, left hand only, right hand only)  • Distance (reach distance between user and board, 40 – 90 centimeters) | *In-game feedback*  • Score (number of buttons pressed including bonus points for streaks which add up dependent on the number of buttons hit in a row, you lose the streak when hitting a button with the wrong hand)  • Number of buttons pressed in a streaks (i.e., pressing two or more buttons in a row with the prescribed hand)    *Post-game feedback*  • Score    *Web portal feedback*  • Prescribed and active minutes  • Number of buttons pressed  • Score |
| **Basketballl**    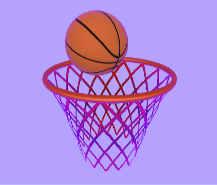 | A sit-to-stand rehabilitation exercise to train dynamic balance and lower-limb muscle strength.    The goal is to score as many points as possible by completing sit-to-stand or squat-to-stand movements (a game-mode setting) to spawn a set of three basketballs, and throw them into the hoop. | • Duration of the exercise: (1 - 10 minutes)  • Difficulty level (number of required sit-to-stands or squats per three balls, 1 - 9 sit-to-stand/squat)  • Game mode (sit-to-stand, squat mode)  • Rhythmic music (on, off) | *In-game feedback*  • Number of prescribed and performed sit-to-stands or squats  • Number of basketballs scored  • Score (number of sit-to-stands or squats plus two times the number of basketballs scored)    *Post-game feedback*  • Score    *Web portal feedback*  • Prescribed and active minutes  • Number of sit-to-stands or squats  • Basketballs scored  • Score |
| **Puzzle Walk**    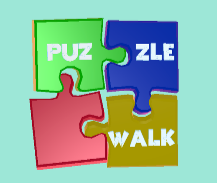 | A goal-directed walking rehabilitation exercise to train gait, dynamic balance, turning, stopping and functional reaching.    The goal is to find puzzle pieces in the room, pick them up by reaching and grabbing them with your hand and then placing them on the easel to complete the puzzle before the time runs out. | • Duration of the exercise (1 - 10 minutes)  • Difficulty level (number of puzzle pieces, 4 – 48 pieces)  • Game mode (puzzle piece height: high, hip, knee, floor) | *In-game feedback*  • Number of prescribed and collected puzzle pieces    *Post-game feedback*  • Score (number of collected puzzle pieces within the set game duration, bonus points for every second left on the clock)    *Web portal feedback*  • Prescribed and active minutes  • Meters walked  • Score |
| **Wobbly Waiter**    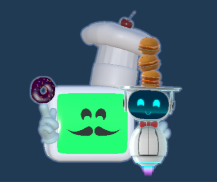 | A cued rehabilitation exercise focusing on walking at a set speed, turning and standing-up/sitting-down up from/in a chair, with an element of cognitive challenge while seated (memory retention).    The goal is to memorize a cafe order from a customer, assemble the order by selecting the buttons with the correct food items in the right sequence, and deliver the order to the customer’s table within a prescribed amount of time, as cued by the waiter’s speed. | • Duration of the exercise (1 -10 minutes)  • Difficulty Level (number of items to remember, 3 – 6 items)  • 10-meter walk test (results)  • Timed Up-and-Go test (results)  • Gait speed adjustment (+/- 25%) | *In-game feedback*  • Cognitive results (number of items correctly remembered)  • Money collected  • Prescribed and performed completion durations    *Post-game feedback*  • Score (money collected)    *Web portal feedback*  • Prescribed and active minutes  • Meters walked  • Score  • Cognitive results  • completion (sub) durations |
| **Strolll AR cues** | | | |
|  | **Description of the cue** | **Setting options** |  |
| **Lines**     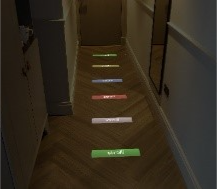 | A series of flat, 2D colored lines on the ground in front of the user to step over. | Color, step length, line width and line thickness. |  |
| **Obstacles**     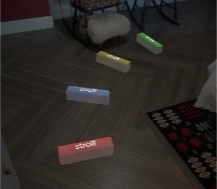 | A series of 3D colored obstacles on the ground in front of the user to step over. | Color, step length, obstacle width, obstacle height, and obstacle thickness. |  |
| **Rhythm** | An audible cue, with different action oriented rhythmic audio sounds for the user to step in time to. | Sound, volume, and speed (beats per minute). |  |
| **Dinosaur footprints**     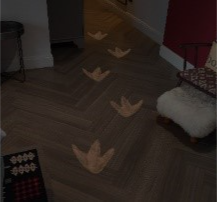 | A series of dinosaur footprints on the ground in front of a user to step on with audible feedback (mud sound). | Step length and step width. |  |

# Supplementary Data Sheet 3. Strolll AR evaluation questionnaire for participants

**Strolll AR evaluation questionnaire**

**Technology acceptance**

The questions below are about the Strolll AR gait-and-balance exercises. For each of the following statements, indicate to what extent you agree with the statement.

|  | **Totally disagree** | **Disagree​** | **Neutral** | **Agree​** | **Totally agree​** |
| --- | --- | --- | --- | --- | --- |
| 1. I now know how to use Strolll AR. |  |  |  |  |  |
| 2. It was easy to use Strolll AR independently. |  |  |  |  |  |
| 3. In case of technical problems with Strolll AR, I could get help from someone. |  |  |  |  |  |
| 4. It was easy to learn how Strolll AR works. |  |  |  |  |  |
| 5. I would like to use Strolll AR regularly if I could keep the glasses. |  |  |  |  |  |
| 6. My living environment is suitable for the use of Strolll AR. |  |  |  |  |  |
| 7. My therapist encouraged the use of Strolll AR throughout the study. |  |  |  |  |  |
| 8. Strolll AR helps me to improve my gait and balance. |  |  |  |  |  |
| 9. Strolll AR helps me to be more physically active. |  |  |  |  |  |
| 10. I find Strolll AR useful to use at home to train gait and balance. |  |  |  |  |  |
| 11. I would continue to train with Strolll AR if I could keep the glasses. |  |  |  |  |  |
| 12. It was clear and understandable how Strolll AR works. |  |  |  |  |  |
| 13. People who are important to me encouraged me to use Strolll AR. |  |  |  |  |  |
| 14. It was easy to get comfortable using Strolll AR. |  |  |  |  |  |
| 15. Using Strolll AR can improve my physical health. |  |  |  |  |  |
| 16. I would like to continue using Strolll AR in the future. |  |  |  |  |  |
| 17. People who influence my behavior encouraged me to use Strolll AR. |  |  |  |  |  |

**Technology use**

1. Do you use a laptop or computer?

- Yes
- Yes, but with help
- No

1. Do you use a smartphone?

- Yes
- Yes, but with help
- No

1. Do you use a tablet?

- Yes
- Yes, but with help
- No

1. How often do you use the internet?

- Rarely/never
- Once a week
- A few times a week
- Once a day
- Several times a day

1. How often do you read or send emails?

- Rarely/never
- Once a week
- A few times a week
- Once a day
- Several times a day

1. How often do you use apps (e.g., weather, navigation, calendar, video calling, internet banking, games)

- Rarely/never
- Once a week
- A few times a week
- Once a day
- Several times a day

**Top-3 barriers and facilitators of Strolll AR**

This top-3 barriers and facilitators is about the Strolll AR gait-and-balance exercises performed at home prescribed and individualized by the therapist. Fill out the top 3 based on what you know about Strolll AR and based on what you have experienced during the study. There are no right or wrong answers. Try to answer as honestly as possible.

What do you think are the **main advantages** of Strolll AR for you as a person with Parkinson’s disease if you could use Strolll AR in the future? Give a **top 3** and rank the advantages in order of importance, with the most important advantage at 1. Give a brief explanation of each advantage.

1. ______________________________________________________________________ Explanation:___________________________________________________________________________________________________________________________________________
2. ______________________________________________________________________ Explanation:___________________________________________________________________________________________________________________________________________
3. ______________________________________________________________________ Explanation:___________________________________________________________________________________________________________________________________________

What do you think are the **main disadvantages** of Strolll AR for you as a person with Parkinson’s disease if you could use Strolll AR in the future? Give a **top 3** and rank the disadvantages in order of importance, with the most important disadvantage at 1. Give a brief explanation of each disadvantage.

1. ______________________________________________________________________ Explanation:___________________________________________________________________________________________________________________________________________
2. ______________________________________________________________________ Explanation:___________________________________________________________________________________________________________________________________________
3. ______________________________________________________________________ Explanation:___________________________________________________________________________________________________________________________________________

**Intervention-specific questions** 
For each of the following statements, indicate to what extent you agree with the statement.

***The questions below are about the*** ***Strolll AR gait-and-balance exercises***

1. To be able to train with Strolll AR, I had to give up other daily activities.

- Totally disagree
- Disagree
- Neutral
- Agree
- Totally agree

If you had to give up any daily activities, please indicate what they were:

____________________________________________________________________________________________________________________________________________________________

1. Training at home with Strolll AR is a good addition to my current (physio)therapy.

- Totally disagree
- Disagree
- Neutral
- Agree
- Totally agree

Can you explain your answer?

____________________________________________________________________________________________________________________________________________________________

1. I enjoyed training with Strolll AR whenever it fitted my schedule.

- Totally disagree
- Disagree
- Neutral
- Agree
- Totally agree

Can you explain your answer?

____________________________________________________________________________________________________________________________________________________________

1. I enjoyed training in my own home.

- Totally disagree
- Disagree
- Neutral
- Agree
- Totally agree

Can you explain your answer?

____________________________________________________________________________________________________________________________________________________________

1. I found the remote supervision of the therapist sufficient to be able to train with Strolll AR.

- Totally disagree
- Disagree
- Neutral
- Agree
- Totally agree

Can you explain your answer? ____________________________________________________________________________________________________________________________________________________________

1. The fact that the therapist can see remotely if I did my exercises at home motivates me to train.

- Totally disagree
- Disagree
- Neutral
- Agree
- Totally agree

Can you please explain your answer? ____________________________________________________________________________________________________________________________________________________________

1. I felt safe while training at home with Strolll AR.

- Totally disagree
- Disagree
- Neutral
- Agree
- Totally agree

Can you explain your answer? ____________________________________________________________________________________________________________________________________________________________

1. During the training with Strolll AR, I was afraid of falling.

- Totally disagree
- Disagree
- Neutral
- Agree
- Totally agree

Can you explain your answer? ____________________________________________________________________________________________________________________________________________________________

***You should answer the question below on a scale of 0-10. A 0 means 'very unlikely' and a 10 means 'very likely'.***

1. How likely are you to recommend the Strolll AR gait-and-balance exercises to a friend (with Parkinson's disease)?

| 0 | 1 | 2 | 3 | 4 | 5 | 6 | 7 | 8 | 9 | 10 |
| --- | --- | --- | --- | --- | --- | --- | --- | --- | --- | --- |

**Very unlikely**                 **Very likely**

***The questions below are about the use of the assistance module or cues. These are for example the visual stripes or rhythms to support walking.***

1. Did you use the cues (for example the lines on the floor) in *Smash!* (boxing exercise)?

- Yes
- No

*If you answered 'No' to question 10, you do not need to answer question 11.*

1. How did the cues help you?

- Supporting freezing (i.e., the feeling of being stuck to the ground)
- Taking bigger steps
- Increasing stability while walking
- Navigating where I needed to go
- Helping me to lift my feet
- Other, namely _________________________________________________________

1. I found the cues during *Smash!* helpful.

- Totally disagree
- Disagree
- Neutral
- Agree
- Totally agree

Can you explain your answer? ____________________________________________________________________________________________________________________________________________________________

*Cue Challenge is the exercise where you walked with different types of cues.*

1. Did you play *Cue Challenge* at home?

- Yes
- No

*If you answered 'No' to question 13, you do not need to answer question 14.*

1. How did *Cue Challenge* help you?

- Supporting freezing (i.e., the feeling of being stuck to the ground)
- Taking bigger steps
- Increasing stability while walking
- Navigating where I needed to go
- Helping me to lift my feet
- Other, namely _________________________________________________________

1. I found playing *Cue Challenge* useful.

- Totally disagree
- Disagree
- Neutral
- Agree
- Totally agree

Can you explain your answer? ____________________________________________________________________________________________________________________________________________________________

# Supplementary Data Sheet 4. Strolll AR evaluation questionnaire for therapists

**Strolll AR evaluation questionnaire**

**Technology Acceptance Questionnaire**

The questions below are about Strolll AR platform. For each of the following statements, indicate to what extent you agree with the statement.

|  | **Totally disagree** | **Disagree​** | **Neutral** | **Agree​** | **Totally agree​** | **n.a.** |
| --- | --- | --- | --- | --- | --- | --- |
| 1. I now know how to use Strolll AR |  |  |  |  |  |  |
| 1. It was easy to independently use Strolll AR |  |  |  |  |  |  |
| 1. In case of technical problems with Strolll AR, I could get help from someone |  |  |  |  |  |  |
| 1. It was easy to learn how Strolll AR works |  |  |  |  |  |  |
| 1. I would like to use Strolll AR regularly if I could keep the glasses |  |  |  |  |  |  |
| 1. My work environment is suitable for the use of Strolll AR. |  |  |  |  |  |  |
| 1. My supervisor encouraged the use of Strolll AR throughout the study. |  |  |  |  |  |  |
| 1. The Strolll AR training helps my patients to improve their gait and balance |  |  |  |  |  |  |
| 1. The Strolll AR training helps my patients to be more physically active |  |  |  |  |  |  |
| 1. I find Strolll AR training useful to use as home training for gait and balance |  |  |  |  |  |  |
| 1. I would continue to give the Strolll AR training if I could keep the glasses |  |  |  |  |  |  |
| 1. It was clear and understandable how the Strolll AR works |  |  |  |  |  |  |
| 1. People who are important to me encouraged me to use Strolll AR |  |  |  |  |  |  |
| 1. It was easy to get comfortable using Strolll AR. |  |  |  |  |  |  |
| 1. Using Strolll AR can help my patients to improve their physical health |  |  |  |  |  |  |
| 1. I would like to continue using Strolll AR in the future |  |  |  |  |  |  |
| 1. People who influence my behavior encouraged me to use Strolll AR |  |  |  |  |  |  |

**Top-3 barriers and facilitators of Strolll AR**
This top 3 questionnaire is about the Strolll AR, an augmented reality exercise program for people with Parkinson's, which you have given to your patients as a practitioner in the past period. By Strolll AR we mean the exercise program as a whole, namely the augmented reality home training with the various exercises and the web portal that you have used as a practitioner. This short top-3 questionnaire is administered at two moments: you completed the questionnaire prior to the start of the CAPARE project (after the training) and you are completing the questionnaire now, after completion of the CAPARE project in your practice.

You may mention the same advantages or disadvantages that you wrote down during the first questionnaire, if they still apply. In this new top 3 of advantages and disadvantages, clearly indicate whether the advantage or disadvantage you mentioned earlier still applies. Fill in the top 3 based on what you know about Strolll AR and based on what you experienced during the training. There are no right or wrong answers. Try to answer as honestly as possible.

What do you think are the **main advantages of Strolll AR** **for you as a healthcare provider and/or for people with Parkinson's** if the intervention is implemented in clinical practice? Make a **top 3** and indicate for each advantage whether it is an advantage for you as a healthcare provider or an advantage for someone with Parkinson's. Put the advantage in order of importance, with the most important benefit at number 1. So you may mention benefits for you (as a healthcare provider) as well as for people with Parkinson's, but you don't have to. The point is that you name the (in your opinion) most important advantages. Give a brief explanation for each advantage.

1. _____________________________________________________________________

Explanation:____________________________________________________________________________________________________________________________________________

This is a benefit that I also mentioned in the top 3 prior to the start of Project CAPARE: Yes / No (cross out what does not apply)

1. _____________________________________________________________________

Explanation:____________________________________________________________________________________________________________________________________________

This is a benefit that I also mentioned in the top 3 prior to the start of Project CAPARE: Yes / No (cross out what does not apply)

1. _____________________________________________________________________

Explanation:____________________________________________________________________________________________________________________________________________This is a benefit that I also mentioned in the top 3 prior to the start of Project CAPARE: Yes / No (cross out what does not apply)

What are the **main disadvantages** of Reality **for you as a healthcare provider and/or for people with Parkinson's** if the intervention were to be implemented in clinical practice? Make a **top 3** and clearly indicate for each disadvantage whether it is a disadvantage for you as a healthcare provider or a disadvantage for someone with Parkinson's. Put the disadvantages in order of importance, with the most important disadvantage at 1. You may therefore mention disadvantages for you (as a healthcare provider) as well as for people with Parkinson's, but you do not have to. The point is that you name the (in your opinion) most important disadvantages. Give a brief explanation for each disadvantage.

1. _____________________________________________________________________

Explanation:____________________________________________________________________________________________________________________________________________
This is a disadvantage that I also mentioned in the top 3 prior to the start of Project CAPARE: Yes / No (cross out what does not apply)

1. _____________________________________________________________________

Explanation:____________________________________________________________________________________________________________________________________________This is a disadvantage that I also mentioned in the top 3 prior to the start of Project CAPARE: Yes / No (cross out what does not apply)

1. _____________________________________________________________________

Explanation:____________________________________________________________________________________________________________________________________________This is a disadvantage that I also mentioned in the top 3 prior to the start of Project CAPARE: Yes / No (cross out what does not apply)

**Intervention-specific questions**

***You should answer the question below on a scale of 0-10. A 0 means 'very unlikely' and a 10 means 'very likely'.***

1. How likely are you to recommend the Strolll AR gait-and-balance exercises to a colleague?

| 0 | 1 | 2 | 3 | 4 | 5 | 6 | 7 | 8 | 9 | 10 |
| --- | --- | --- | --- | --- | --- | --- | --- | --- | --- | --- |

**Very unlikely** **Very likely**

***The questions below are about the use of the assistance module, or cues. These are for example the visual stripes or sound rhythms to support walking.***

1. Did you prescribe the cues (for example the lines on the floor) for *Smash!* (boxing)?

- Yes
- No

*If you answered 'No' to question 2, you do not need to answer question 3.*

1. I found the cues during *Smash!* to be of added value for people with Parkinson's disease.

- Totally disagree
- Disagree
- Neutral
- Agree
- Totally agree

Can you explain your answer to the above question? ___________________________________________________________________________________________________________________________________________________________________________________________________________________________________________________

*CueChallenge is the game where participants walk (laps) with different types of cues.*

1. Do you have *CueChallenge* prescribed at home?

- Yes
- No

*If you answered 'No' to question 4, you do not need to answer question 5.*

1. I found the cues during *Smash!* to be of added value for people with Parkinson's disease.

- Totally disagree
- Disagree
- Neutral
- Agree
- Totally agree

Can you explain your answer to the above question? ___________________________________________________________________________________________________________________________________________________________________________________________________________________________________________________

***The question below is about the therapy you typically provide to your patients with Parkinson's disease.***

1. Do you typically provide exercises for patients with Parkinson's disease to perform independently at home as part of their therapy?

- Yes
- No

Can you describe what kind of exercises you give to your patients? ___________________________________________________________________________________________________________________________________________________________________________________________________________________________________________________

***If you answered 'no' to question 6, you do not need to answer questions 7 and 8.***

1. In what form do you usually prescribe these exercises? For example, on paper, via a mobile app or Youtube video. ____________________________________________________________________________________________________________________________________________________________________________________________________________________________________
2. How often do you typically ask your patients with Parkinson's to do these exercises? ____________________________________________________________________________________________________________________________________________________________________________________________________________________________________

# Supplementary Data Sheet 5. Randomization Matlab script

N = 58:2:100; %number of participants

r = 20; % number of randomisations

struct_random = struct();

rng("shuffle");

for ii = 1:length(N)

A = [ones((N(ii)/2),1); zeros((N(ii)/2),1)];

for i=1:r

struct_random(ii).randomisatie(:,i)=A(randperm(length(A)));

struct_random(ii).creation_time = datetime('now');

end

end

%%

password = uint8('password12345678'); % insert a password which has to be 16 characters exact

% Encryption

dataJson = jsonencode(struct_random); % Convert struct to JSON string

dataBytes = uint8(dataJson); % Convert the JSON string to a byte array

% Create AES cipher instance

cipher = javax.crypto.Cipher.getInstance('AES/ECB/PKCS5Padding'); % Specify padding

keySpec = javax.crypto.spec.SecretKeySpec(password, 'AES');

cipher.init(javax.crypto.Cipher.ENCRYPT_MODE, keySpec);

% Encrypt the data

encryptedData = cipher.doFinal(dataBytes);

% Save encrypted data to MAT file

save('encryptedData.mat', 'encryptedData');

disp('Data encrypted and saved using AES.');
